# Med. Society at Penn

**Published:** 1889-09

**Authors:** 


The Medical Society at Penn., appointed to meet on Sept. 30, of present year, has been postponed, on account of the Johnstown and other disasters, until the 2d Tuesday in June, 1890.

